# The ocular hypotensive effect of saffron extract in primary open angle glaucoma: a pilot study

**DOI:** 10.1186/1472-6882-14-399

**Published:** 2014-10-15

**Authors:** Mohammad Hossein Jabbarpoor Bonyadi, Shahin Yazdani, Saeed Saadat

**Affiliations:** Department of Ophthalmology, Gonabad University of Medical Sciences, Gonabad, Iran; Center of Excellence for Biodiversity, Faculty of Natural Sciences, University of Tabriz, Tabriz, Iran; Ophthalmic Research Center, Shahid Beheshti University of Medical Sciences, Tehran, Iran; School of Medicine, Shahroud University of Medical Sciences, Shahroud, Iran

**Keywords:** Open angle glaucoma, Saffron extract, Hypotensive effect, Antioxidative effect

## Abstract

**Background:**

The progressive nature of glaucoma and its growing incidence make its therapy an important target for research. The role of oxidative damage in the pathogenesis of glaucoma makes antioxidants such as saffron extract an attractive target for potential clinical use. Herein, we evaluate the effect of aqueous saffron extract on intraocular pressure (IOP) in eyes with primary open-angle glaucoma (POAG).

**Methods:**

Thirty-four eyes of 34 clinically stable POAG patients receiving treatment with timolol and dorzolamide eye drops were enrolled in this prospective, comparative, randomized interventional pilot study. Eligible subjects were randomized to receive 30 mg/day aqueous saffron extract orally (17 subjects, 17 eyes) or placebo (17 subjects, 17 eyes) for one month as an adjunct to timolol and dorzolamide. Following treatment, both study groups entered a one-month wash-out period. The main outcome measure was IOP during treatment and after the wash-out period.

**Results:**

Mean baseline IOP was 12.9 ± 3.7 versus 14.0 ± 2.5 mmHg in the saffron and control groups, respectively (p = 0.31). After three weeks of treatment, IOP was significantly decreased to 10.9 ± 3.3 mmHg in the saffron group as compared to 13.5 ± 2.3 mmHg in the control group (p = 0.013). At four weeks, IOP was still significantly lower in the saffron group (10.6 ± 3.0 versus 13.8 ± 2.2 mmHg, p = 0.001). At the end of the wash-out period, IOP was 12.9 ± 3.0 in the saffron group versus 14.2 ± 2.0 mmHg in the control group (p = 0.175). None of the patients experienced side effects during the study and wash-out period.

**Conclusions:**

Oral aqueous saffron extract seems to exert an ocular hypotensive effect in primary open-angle glaucoma. This effect became evident after three weeks of therapy.

The current study was registered at the International Clinical Trials Registry Platform (ICTRP) as IRCT201201278832N1.

## Background

Glaucoma is the most common progressive optic neuropathy [1] and the most important risk factor for development of glaucomatous optic neuropathy is elevated intraocular pressure (IOP) [2]. Abnormal resistance to the outflow of aqueous humor through the trabecular meshwork (TM) is the key pathogenic factor in primary open angle glaucoma (POAG) [3].

Oxidative stress has been suggested to play a role in POAG and the trabecular meshwork is the most sensitive anterior chamber structure to oxidative stress [4–8]. The important role of oxidative damage in the pathogenesis of POAG makes it an important therapeutic target and antioxidative therapy has been shown to entail an ocular hypotensive effect against dexamethasone induced IOP elevation [9].

Saffron, derived from the pistils of *Crocus sativus*, contains high concentrations of crocin and crocetin, which are carotenoid derivatives. This plant belongs to the Iridaceae family. Multiple divalent carbon bonds in saffron compounds confer its powerful radical scavenging, antioxidative and anti-tumor properties [10–13]. Saffron has been widely used for many years in traditional Asian medicine and in Persian medicine in particular to treat depression [14].

The purpose of this pilot study was to investigate the possible ocular hypotensive effect of high dose oral aqueous saffron extract on IOP in POAG. To the best of our knowledge this is the first report on this subject.

## Methods

### Setting

This randomized study was performed at the department of Ophthalmology, 22nd of Bahman Hospital, Gonabad, Iran in collaboration with the Ophthalmic Research Center, Shahid Beheshti University of Medical Sciences, Tehran, Iran. Consecutive POAG patients were recruited from the ophthalmology clinic and assessed for eligibility. Patients who met the inclusion criteria entered our pre-study observation period (February 2012- March 2013) during which stability of glaucoma was confirmed (for at least 6 months) while the subjects were receiving topical medications. Eventually 50 eyes of the same number of stable POAG patients were enrolled for intervention (April 2013). This study was registered at the International Clinical Trials Registry Platform (ICTRP) as IRCT201201278832N1.

### Inclusion and exclusion criteria

Patients with POAG older than 50 years of age who were under treatment with topical timolol 0.5% twice daily and dorzolamide 2% three times daily and were judged to have stable glaucoma control for at least 6 months based on a series of visual field and optic nerve head examinations were considered for enrollment as stated earlier.

Exclusion criteria consisted of prior intraocular surgery, glaucoma other than POAG, visual acuity less than 5/10, advanced glaucoma (equal or more than 90 percent vertical cupping), history of saffron hypersensitivity, intestinal absorption abnormalities and smoking. In the case of bilateral involvement, only the right eye was included. None of the patients were taking medications (e.g., chloroquine) known to interfere with carotenoid absorption. Patients with central corneal thickness (CCT) values less than 500 or more than 540 microns were also excluded.

During the study period, enrolled subjects would be eliminated from the study if they failed to attend regular follow-up sessions, maintain a regular regimen of topical glaucoma medication or saffron/placebo, or consumed a systemic medication with a known effect on IOP.

### Informed consent and ethical issues

The study adhered to the tenets of the Declaration of Helsinki and was approved by the Ethics Committee of Gonabad Medical University. Prior to enrollment, potential advantages and risks of therapy were discussed with the patients and written informed consent was obtained from all participants. Consent forms contained possible side effects of high dose oral saffron including anxiety, appetite changes, nausea, headache, dry mouth, constipation, allergic reactions and jaundice.

### Pre-intervention evaluations and follow-up

All participants underwent a detailed baseline ophthalmic examination including slitlamp biomicroscopy, gonioscopy, dilated lens and optic nerve head examination and IOP measurement using Goldmann applanation tonometry in addition to corneal pachymetry (OcuScan, Alcon Laboratories, Irvine, California, USA). For all cases and control patients, IOP was measured between 9 to 10 AM at baseline, at 1, 3 and 4 weeks following initiation of intervention, and at the end of the wash-out period.The list of side effects as mentioned in Informed Consent and Ethical Issues section was checked in each session. Every patient was asked and examined according to mentioned list and also asked about any other symptom in each exam session to reveal possible side effects.

### Randomization and masking

Patients were randomly assigned to the saffron regimen or placebo (simple randomization in two parallel groups). Both capsules were identical regarding shape, color and size. Participants were kept masked to the intervention and outcome measures, the ophthalmologist evaluating the outcome measure and the statistician involved in data analysis were also unaware of patient identity and study group. Eligible patients were introduced to our secretary who randomly selected one sealed, opaque envelope with 28 capsules containing saffron extract or placebo for each patient according to the randomization sheet.

The allocated group of each patient was registered and concealed up to the end of the study period and revealed only after final data analysis.

### Intervention

*Crocus sativus L.* stigmas were collected from Gonabad (East Sorkhfam Saffron Co. Gonabad, Khorasan, Iran). Using the maceration method, saffron capsules containing thirty milligrams of the concentrated powder were prepared [12]. Starch powder was used with the same weight inside identical capsules for the placebo group. Capsule preparation for both study groups was performed by Genaati pharmacy, Gonabad, Khorasan, Iran. Study subjects were randomly assigned to oral saffron extract (30 mg/day) versus placebo for a one month period while topical timolol and dorzolamide were continued throughout the randomization process. Following treatment, both groups entered a one-month wash-out period, during which all patients stopped ingestion of saffron/placebo capsules but continued their glaucoma medications as before. At the end of the wash-out period IOP measurement (using Goldmann applanation tonometry) and ophthalmologic evaluation (by a masked ophthalmologist MHJB) was repeated. During the study period patients were asked about and examined for possible side effects of saffron.

### Outcome measures

The main outcome measure of the study was IOP.

### Statistical methods

In this pilot RCT we enrolled 25 POAG patients in each group. Means and standard deviations were used for description of study parameters. Statistical analysis was performed by a biostatistician using SPSS software version 17.0 (SPSS Co., Chicago, IL, USA) who was unaware of study group identity. Mean CCT and mean IOP differences between study and control groups were analyzed individually by independent student t-test. The Mann-whitney test was employed to compare mean vertical cup disc ratio between the two groups because of non-normal distribution of this variable.

## Results

Initially 50 subjects fulfilled the eligibility criteria and were enrolled but a total of 16 subjects were eliminated from the study. The excluded patients in the saffron group consisted of 5 patients with incomplete follow-up and 3 patients who used their capsules irregularly. Excluded patients in the placebo group consisted of 6 patients with incomplete follow-up, one patient with irregular capsule consumption and another subject who started a systemic B blocker during the study period.

Eventually a total of 34 randomized subjects including 17 patients (7 female and 10 male individuals) in the saffron group and 17 other patients (6 female and 11male individuals) in the placebo group completed all follow-up sessions. None of the enrolled patients experienced side effects of saffron during the study or the wash-out period.

**Figure 1:**
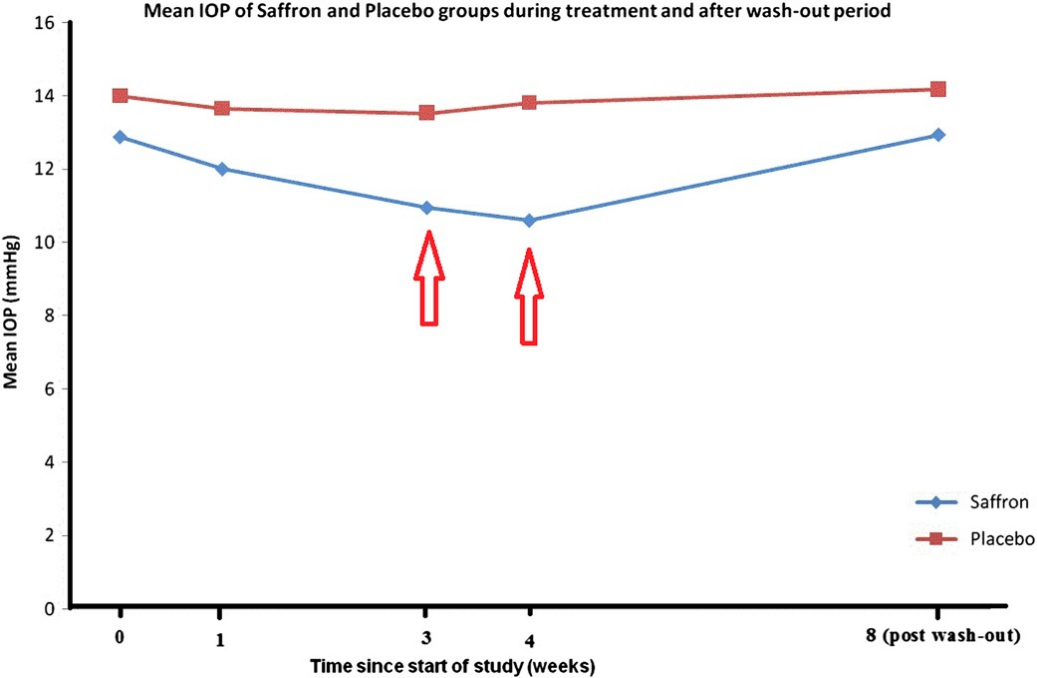
**Mean IOP change during 4 weeks after initiation of saffron or placebo in addition to conventional timolol and dorzolamide therapy in primary open angle glaucoma and after one month of wash-out (Arrows indicate where mean IOP levels were significantly different between the two study groups).**

Mean patient age was 66.3 ± 9.5 (range 51 to 79) years versus 67.6 ± 8.3 (range 53 to 77) years in the saffron and placebo groups, respectively (p = 0.69). CCT was 518 ± 13.1 microns in the saffron group versus 519 ± 14.7 microns in the control group (p = 0.98). Mean vertical cup disc ratio was 0.68 ± 0.10 in the saffron group and 0.71 ± 0.08 in the placebo group (P = 0.33). Mean baseline IOP was 12.9 ± 3.7 (range 7 to 19) mmHg in the saffron group and 14.0 ± 2.5 (range 9 to 18) mmHg in the control group (95% confidence interval [CI] for mean difference:-1.1 ± 2.2 mmHg, p = 0.31).Baseline features of enrolled patients are summarized in Table 1.One week after intervention, IOP was 12.0 ± 3.3 (range 8 to 17) mmHg in the saffron group versus 13.6 ± 2.6 (range 9 to 18) mmHg in the control group (95% CI for mean difference: -1.65 ± 2.05 mmHg, p = 0.114). At three weeks, IOP reached 10.9 ± 3.3 (range 6 to 17) mmHg in the saffron group versus 13.5 ± 2.3 (range 9 to 17) mmHg in the control group (95% CI for mean difference; -2.6 ± 2.00 mmHg, p = 0.013). At four weeks IOP was 10.6 ± 3.0 (range 6 to 16) mmHg in the saffron group versus 13.8 ± 2.2 (range 10 to17) mmHg in the control group (95% CI for mean difference: -3.25 ± 1.85 mmHg, p = 0.001). At the end of the wash-out period, IOP was 12.9 ± 3.0 (range 8 to 18) mmHg in the saffron group and 14.2 ± 2.0 (range 11 to 18) mmHg in the control group (95% CI for mean difference: -1.20 ± 1.80 mmHg, p = 0.175) (Figure 1).

**Table 1:** **Baseline characteristics of patients**

	Saffron group	Placebo group	
Male	10	11	p^β^ = 0.72
Female	7	6	
Age	66.3 ± 9.5	67.6 ± 8.3	p* = 0.69
CCT	518 ± 13.1	519 ± 14.7	p* = 0.98
vertical cup disc ratio	0.68 ± 0.10	0.71 ± 0.08	P® = 0.33
VF MD	-5.47 ± 1.5	-4.99 ± 1.9	p* = 0.416

CCT: central corneal thickness, VF MD: visual field defect mean deviation.

β: based on Chi-square test.

*: based on t-test.

®: based on Mann-whitney test.

## Discussion

Saffron has been widely used for many years in traditional Persian medicine to treat depression [14] and its crude extract has recently been shown to prevent tumor formation [13] and selenite-induced cataracts [15].

Saffron belongs to the Iridaceae family and its variants are not of the same quality and strength. Strength is related to several factors including the amount of styles picked along with the red stigma. Grades of Iranian saffron are: “Sargol” (red stigma tips only, premium grade), “Pushal” or “Pushali” (red stigmas plus some yellow style, lower strength), “Bunch” saffron (red stigmas plus large amounts of yellow style, presented in a tiny bundle) and “Konge” (yellow style only, claimed to have aroma but with very little, if any, coloring potential). In our study only premium Sargol grade fresh saffron was used by East Sorkhfam Saffron Co. for capsule preparation.

Antioxidants, ingested through diet and supplements, have been shown to be beneficial in terms of reducing the risk of multiple ocular diseases including age-related macular degeneration (based on the Age-Related Eye Disease Study, AREDS) [16] and cataracts [17].

A number of studies have strongly suggested the role of oxidative stress in POAG [4–8, 18, 19] and there is increasing evidence that oxidative stress also has an important role in progression of glaucomatous damage [20]. The TM is the most sensitive anterior chamber tissue to oxidative stress. [4] Light induced formation of oxidative radicals may target the TM and contribute to the pathogenesis of glaucoma [4, 21]. Disturbed TM cell homeostasis by oxidative stress may lead to cellular loss and structural alterations in its extracellular matrix resulting in impaired aqueous humor outflow and thereby an increase in IOP [22, 23]. In addition to the observed effect of saffron extract regarding IOP reduction, its possible role as antioxidant-neuroprotective agent [24] may further provide protection against glaucomatous optic neuropathy.

Other agents with antioxidant properties have been used for treatment of glaucoma. Ginkgo biloba, similar to saffron, has antiapoptotic and antioxidant capacities [25, 26] and has demonstrated desirable effects on steroid-induced ocular hypertension. In a rabbit model, Jia et al [9] demonstrated that topical Ginkgo biloba extract 4 times daily could suppress dexamethasone induced IOP elevation after 14 days of treatment. In another in vitro study, use of vitamin E as an antioxidant agent was shown to reduce the production of reactive oxygen species and inhibit cell death in TM cell cultures derived from glaucomatous patients [27]. Oxidative stress, in vitro, has been shown to result in mitochondrial and cellular dysfunction in the TM resulting in trabecular cell apoptosis [27, 28]. Therefore antioxidants seems to exert both a short term effect in terms of rehabilitating damaged but still functional TM*,* and a long term benefit by reducing apoptosis. The ocular hypotensive effect of saffron extract in our study could be due to the short term effect.

The current study showed that oral aqueous saffron extract had an ocular hypotensive effect in POAG patients receiving treatment with timolol and dorzolamide. This effect became evident three weeks after therapy and was accentuated at four weeks.Employing a washout period, IOP returned to pre-treatment levels after one month of discontinuation. (Figure 1) The observed reduction in IOP may be due to antioxidant carotenoid derivatives present in saffron extract, mainly crocin and crocetin.

In a recently published epidemiologic study in an African-American population it has been shown that carotenoids seem to display a protective effect against glaucoma [29]. In contrast, Kang et al reported no association between higher intake of various antioxidants and glaucoma [30]. Such differences in the correlation between glaucoma risk and carotenoids may be due to differences in study population, age, eating habits and food preparation methods. It has also been shown that genetic predisposition to oxidative stress may determine susceptibility to glaucoma [18] and the response to antioxidants as ocular hypotensive agents seems to be influenced by genetic background.

In the current study, we selected POAG patients whose IOP was controlled with two topical medications. We assume that regarding the role of oxidative damage in the pathogenesis of glaucoma, our results may be generalizable for POAG of different severity levels and possibly other types of glaucoma. Although our observations are promising, there are limitations which have to be kept in mind; regular high dose crude oral saffron may not be a feasible treatment option for glaucoma while small sample size and short term follow-up in this pilot study are restrictions of our study. Future studies with larger sample size and longer follow-up on different types glaucoma with varying severity could verify our results.

Saffron shown to have a very low toxicity in *in vivo* studies and daily doses of up to 1.5 gr are thought to be safe. Toxic effects are reported with 5 gr and above, with a lethal dose of approximately 20 gr and it could induce abortion in doses >10 gr. The colored constituents may accumulate in sclera, skin and may thus mimic icteric complaints. Nausea was described in doses between 1.2 gr and 2 gr followed by vomiting, diarrhea and bleeding [31]. Since the dose shown to be efficacious in depression trials corresponded to approximately 30 mg of saffron, there is a large safety margin [32]”.

## Conclusions

In summary, this pilot study revealed an ocular hypotensive effect from high dose oral aqueous saffron extract in POAG patients when the supplement was added to conventional timolol and dorzolamide treatment. To the best of our knowledge this is the first study reporting the effect of saffron on IOP. This early ocular hypotensive effect, similar to its influence in early age-related macular degeneration, [33] indicates that high dose systemic saffron may entail a therapeutic effect.

It must be kept in mind that high dose antioxidants may have paradoxical pro-oxidative effects exemplified by an increased risk of lung cancer with higher dietary intake of carotene [34, 35]. Whether topically administered saffron compounds may also reduce IOP, requires further studies. Reaching a topical saffron formulation necessitates preparing a topical solution with reduced coloring capacity and minimal sensitizing side effects.

## Abbreviations

POAG: Primary open angle glaucoma
IOP: Intraocular pressure.
